# A Novel Antifungal Actinomycete *Streptomyces* sp. Strain H3-2 Effectively Controls Banana Fusarium Wilt

**DOI:** 10.3389/fmicb.2021.706647

**Published:** 2021-08-23

**Authors:** Niexia Zou, Dengbo Zhou, Yinglong Chen, Ping Lin, Yufeng Chen, Wei Wang, Jianghui Xie, Mingyuan Wang

**Affiliations:** ^1^Institute of Horticultural Science and Engineering, Huaqiao University, Xiamen, China; ^2^Key Laboratory of Biology and Genetic Resources of Tropical Crops, Ministry of Agriculture, Institute of Tropical Bioscience and Biotechnology, Chinese Academy of Tropical Agricultural Sciences, Haikou, China; ^3^School of Agriculture and Environment, The UWA Institute of Agriculture, The University of Western Australia, Perth, WA, Australia

**Keywords:** *Streptomyces* sp., banana fusarium wilt, antifungal mechanism, pot experiment, GC-MS, biocontrol

## Abstract

Banana Fusarium wilt disease caused by *Fusarium oxyspoum* f. sp. *cubense* (Foc) seriously threatens the banana industry. Foc tropical race 4 (Foc TR4) can infect almost all banana cultivars. Compared with traditional physical and chemical practices, biocontrol strategy using beneficial microbes is considered as an environmentally sound option to manage fungal disease. In this study, a strain, H3-2, isolated from a non-infected banana orchard, exhibited high antifungal activity against Foc TR4. According to its morphological, physiological, and biochemical characteristics, the strain H3-2 was identified as *Streptomyces* sp. and convinced by the polymorphic phylogenic analysis of 16S *rRNA* sequences. Extracts of the strain H3-2 suppressed the growth and spore germination of Foc TR4 *in vitro* by destroying cell membrane integrity and mycelial ultrastructure. Notably, the strain and its extracts showed broad-spectrum antifungal activity against the selected seven fungal phytopathogens. Fourteen chemical compounds in the extracts were identified by gas chromatography–mass spectrometer (GC-MS), primarily phenolic compounds. Additional pot inoculation experiment demonstrated that the fermentation broth of the strain H3-2 promoted the growth of banana seedlings by efficiently inhibiting the spread of banana Fusarium wilt disease. This study demonstrated the potential application of the novel *Streptomyces* sp. H3-2 for the management of banana Fusarium wilt.

## Introduction

Banana (*Musa* spp.) is one of the most important fruit crops among the world’s top 10 staple foods in terms of production and trade (Dita et al., 2018; Xu et al., 2020). However, Fusarium wilt disease, known as Panama disease, seriously causes a substantial loss in the banana industry (Dale et al., 2017). Banana Fusarium wilt is a soil-borne fungal pathogen, which is caused by *Fusarium oxysporum* f. sp. *cubense* (Foc). The Foc tropical race 4 (Foc TR4) strain can survive for more than 30 years in soil and infects over 80% of banana cultivars (Zhang et al., 2019). Currently, physical and chemical methods are not effective in controlling the spread of banana Fusarium wilt disease. Additionally, chemical application can induce the development of resistant strains and cause environmental pollution (Kanini et al., 2013). Practical management, including the isolation of infested areas, remotion of infected plants, and disinfection of farm instruments, slows the spread of Foc to a certain extent (Dale et al., 2017). Biological control using functional microbes is considered to be an economically and environmentally friendly method to manage fungal phytopathogens (Abbas et al., 2020).

Actinobacteria belonging to Gram-positive bacteria have a great biosynthetic potential to produce large amounts of bioactive secondary metabolites (Jose et al., 2021). Some metabolites with novel structure and remarkable biological activity are widely used in agriculture, industry, and medicine (Uyeda et al., 2001; Kurniawati et al., 2016; Li et al., 2021a). Nearly 80% of antibiotics are produced by actinomycetes, such as *Streptomyces* (Yun et al., 2018). Many insecticides, bioherbicides, and antifungal agents are also made from *Streptomyces* (Barka et al., 2016). For example, ivermectin, a true success story in terms of anthelmintics killing a variety of parasites and insects, is a dehydro derivative of avermectin produced by *Streptomyces avermitilis* (Omura and Crump, 2014, 2017). Mildiomycin, a nucleoside fungicide isolated from the secondary metabolites of *Streptomyces rimofaciens*, has potent bioactivity against powdery mildews on cucumber or pumpkin (Feduchi et al., 1985; Huang et al., 2010; Zhao et al., 2016). Our previous study also showed that extracts of *Streptomyces* sp. H4 had strong inhibitory activity against *Colletotrichum fragariae* during strawberry fruit storage stage (Li et al., 2021a). *Streptomyces* sp. JBS5-6 also exhibits strong antifungal activity against Foc TR4 (Jing et al., 2020). However, these functional microbes for controlling plant diseases are limited by their different growth conditions or poor activity in a complex environment (Saravanan et al., 2003). The isolation and screening of broad-spectrum and highly efficient antagonistic agents are still necessary (Chen et al., 2018).

In this study, a novel strain H3-2 belonging to the genus *Streptomyces* was isolated from the rhizosphere of banana plantations, and its broad-spectrum antifungal activity was discovered. The morphological, physiological, and biochemical characteristics of *Streptomyces* sp. H3-2 were analyzed. The effects of its extracts on mycelial growth, morphology, and cell ultrastructure and the spore germination of Foc TR4 were also detected. In addition, the biocontrol ability of *Streptomyces* sp. H3-2 was further investigated in a pot experiment. The aim of this study was to excavate important actinobacterium resources to manage fungal phytopathogens. These findings will be helpful in searching novel natural compounds with high antifungal activities for the banana industry.

## Materials and Methods

### Screening of Antifungal Actinomycetes

Actinomycetes were isolated from the rhizosphere of a banana orchard, where no disease symptoms of banana Fusarium wilt were recorded for more than 10 years in Lingao County, Hainan Province, China (19°47′1″N and 105°51′17″E). Isolated strains were preserved on ISP2 medium slants at 4°C in the Key Laboratory of Biology and Genetic Resources of Tropical Crops, Ministry of Agriculture, Institute of Tropical Bioscience and Biotechnology, Chinese Academy of Tropical Agricultural Sciences, Haikou, China. To screen the actinomycetes with antifungal activity against Foc TR4, a plate confrontation method on the potato dextrose agar (PDA) medium was performed *in vitro* according to the description of Li et al. (2021). The inhibition rates of fungal radial growth were calculated using the formula: Inhibition rates = [(A−B)/(A−0.5)] × 100, where A and B are the mean colony diameters (cm) of phytopathogenic fungi in the control and treatment groups, respectively.

### Identification of Strain H3-2

A H3-2 strain was identified by combining morphological, physiological, and biochemical characteristics (Qi et al., 2019). For molecular identification, the 16S *rRNA* gene sequence was amplified by polymerase chain reaction (PCR) using the prokaryotic universal primers (27F: 5′-AGTT TGATCMTGGCTCAG-3′ and 1492R: 5′-GGTTACCTTGTTACGACTT-3′) (Qi et al., 2019). The thermocycling condition for PCR included denaturation at 95°C for 3 min, followed by 32 cycles of denaturation at 94°C for 30 s, annealing at 55°C for 1 min, extension at 72°C for 2 min, and final extension at 72°C for 10 min in the Veriti thermal cycler (Applied Biosystems, Carlsbad, CA, United States). The amplified product was analyzed in 1.2% (w/v) of agarose gel electrophoresis and sequenced by Sangon Biotech Co., Ltd. (Shanghai, China). To perform a phylogenetic analysis, the 16S *rRNA* gene sequence was aligned against the NCBI GenBank entries using the BLAST algorithm^1^ and the EzBioCloud database^2^ to obtain the homology sequences. The phylogenetic tree was constructed using the neighbor-joining method of MEGA version 7.0.

### Isolation of Stain H3-2 Crude Extracts

The H3-2 strain was cultured on ISP2 solid medium for 7 days at 28°C. A single colony was selected and inoculated into a 250-ml Erlenmeyer flask containing 100 ml of the ISP2 liquid medium at 200 rpm for 4 days at 28°C as a seed suspension. To obtain a mass culture fermentation broth, 20 ml of the seed culture broth was inoculated in 20 flasks (5 L) containing 1 L of sterilized soybean liquid culture medium (SLM, 15 g of soybean powder, 20 g of amylose, 5 g of yeast extracts, 2 g of bacterial peptone, 4 g of NaCl, 4 g of CaCO_3_ per liter of distilled water, pH adjusted to 7.2 with 2 mol/L NaOH), respectively. The flasks were cultured at 200 rpm for 7 days at 28°C. The fermentation broth of the 7-day-old strain H3-2 was centrifuged at 8,000 × *g* for 15 min. The supernatant was concentrated under vacuum at 36°C using a rotary evaporator to obtain crude extracts (Jing et al., 2020).

### Purification of H3-2 Crude Extracts

After filtering with filter paper, crude extracts were eluted using a linear gradient (1 L each) of methanol: deionized water (50:50, 60:40, 70:30, 80:20, and 100:0) on a silica gel column (8.0 inner diameter, 60 cm length) and divided into five fractions. The five evaporated solutions were dissolved into 10% of dimethyl sulfoxide (DMSO) with a final concentration of 20.0 mg/ml, respectively. Then, inhibitory efficiency against Foc TR4 was carried out by a plate diffusion method according to previous reports (Jing et al., 2020; Li et al., 2021b): 20 mg/ml of five crude extract solutions were transferred to 60 ml of the autoclaved PDA medium at 50–55°C; 10% of DMSO was used as a control. A 5-mm-diameter fungal disc of Foc TR4 was inoculated in the center of plate. The inhibition rates of Foc TR4 were calculated at 28°C for 7 days as mentioned above. All experiments were repeated in triplicates.

### Effect of Strain H3-2 Extracts on Mycelial Growth of Foc TR4

Growth inhibition of Foc TR4 was evaluated by mycelial growth rate. Strain H3-2 extracts isolated with 100% of methanol were added to the autoclaved PDA medium and diluted into different concentrations of 0.78, 1.56, 3.12, 6.25, 12.5, 25, 50, 100, and 200 μg/ml, respectively. The consistent concentration of DMSO was used as a negative control in each group. A 5-mm-diameter fungal disc of Foc TR4 was inoculated in the center of plate. The growth diameter of Foc TR4 was measured after 7 days at 28°C. Each treatment contained three replicates. A least square method was used to establish a linear regression equation, namely, a toxicity regression equation (Vanewijk and Hoekstra, 1993). A half maximal effective concentration (EC_50_) value was calculated according to the toxicity regression equation.

### Effect of Strain H3-2 Extracts on Mycelial Morphological Characteristics of Foc TR4

The morphological characteristics of Foc TR4 mycelia treated with H3-2 extracts were assessed by scanning electron microscopy (SEM; TM4000Plus, Hitachi, Japan). The methods of sample preparation and analysis were referred to the publication with a slight modification (Wei et al., 2020). A 5-mm-diameter disk of Foc TR4 was inoculated in the PDA plate containing 4 × EC_50_ of strain H3-2 extracts. A transverse section (1 cm^2^) was cut from the mycelial edges. The corresponding position was selected from the control plate containing an equal volume of DMSO. The hyphal samples were fixed with 2.5% (v/v) of glutaraldehyde at 4°C for 4 h and washed three times with 1.5 ml of phosphate-buffered saline (PBS, 0.1 mol/L) for 15 min. Samples were dehydrated through a gradient ethanol (30, 50, 70,80, 90, 95, and 100%) for 15 min. After replacing ethanol with isoamyl acetate, critical point drying was carried out with carbon dioxide. Finally, the dried samples coated with thin gold were observed under SEM.

### Effect of Strain H3-2 Extracts on Mycelial Cell Ultrastructure of Foc TR4

The ultrastructure of Foc TR4 cells treated with H3-2 extracts was investigated by transmission electron microscopy (TEM, HT7700; Hitachi, Ibaraki, Japan). The methods for collection, fixation, and dehydration of samples were the same as described above. The samples were embedded in the Epon812 resin and then were aggregated at 35°C for 12 h, 45°C for 24 h, and 60°C for 48 h. Then, the embedded material was sliced into 70-nm slices by an ultramicrotome (EM UC6; Leica, Wetzlar, Germany) at room temperature. Subsequently, these sections were double-stained with uranyl acetate and lead citrate solution. After natural drying, the ultrastructure of mycelial cells was detected by TEM.

### Effects of Strain H3-2 Extracts on Foc TR4 Spore Germination

The germination rate of Foc TR4 spores was assayed as reported previously with a minor modification (Li et al., 2021b). Briefly, 45 μl of spore suspension (1.0 × 10^6^ CFU/ml) was completely mixed with an equal volume of strain H3-2 extracts with different concentrations of 1 × EC_50_, 2 × EC_50_, 4 × EC_50_, 8 × EC_50_, and 16 × EC_50_, respectively. The mixture was added into concave slides and was incubated at 28°C for 12 h. The same concentration of DMSO or spore suspension was used as the negative control. All experiments were performed in triplicates. One hundred spores in each slide were observed by an optical inverted microscope (MMI Cellcut Plus; MMI, Glattbrugg, Switzerland). The inhibition efficiency was evaluated using the percentage of spore germination (Wei et al., 2020).

### Assay of a Broad-Spectrum Antifungal Activity of Strain H3-2 and Its Extracts

To test the broad-spectrum antifungal activity of the stain H3-2 and its extracts, seven fungal phytopathogens, including Foc TR4 (ATCC 76255), *F. oxysporum f*. sp. *cucumerinum* (ATCC 204378), *Fusarium graminearum* (ATCCMYA-4620), *Colletotrichum gloeosporioides* (ATCCMYA-456), *Colletotrichum gloeosporioides* (ACCC 36351), *Pyricularia oryzae* (ATCC 52352), *Colletotrichum fragariae* (ATCC 58718), and *Curvularia lunata* (ATCC 12017), were selected from the Key Laboratory of Biology and Genetic Resources of Tropical Crops, Ministry of Agriculture, Institute of Tropical Bioscience and Biotechnology, Chinese Academy of Tropical Agricultural Sciences, Haikou, China. Strain H3-2 was inoculated at four symmetrical sites on the PDA plate. After 24 h at 28°C, a 5-mm-diameter disc of phytopathogenic fungi was added in the center of PDA plate. For further detection of antifungal activity of strain H3-2 extracts, each fungal phytopathogen was inoculated in the center of an autoclaved PDA medium with 200 μg/ml of final extract concentration. Antifungal activity was measured using the mycelial growth method. All experiments were repeated in triplicates.

#### Chemical Constituent Analysis of Strain H3-2 Extracts

The chemical constituent of H3-2 extracts was analyzed by a PerkinElmer 690 GC coupled to a PerkinElmer 8T Single Quadrupole MS. The Elite-5MS capillary column (30 m length, 0.25 mm inner diameter, 0.25 μm film thickness) and helium (1 ml min^–1^) were used as stationary phase and mobile phase, respectively. The splitless mode was employed for injection with injection quantity of 1 μl and injector temperature control of 250°C. The column temperature program was set as follows: initial temperature kept at 60°C for 1 min, increased to 100°C at a rate of 5°C min^–1^ for 5 min, then increased to 250°C at a rate of 10°C min^–1^ for 35 min, programmed to 280°C at a rate of 10°C min^–1^, and finally kept isothermally at 280°C for 25 min. The temperature of transfer line was 280°C, and the temperature of ion source was 240°C. The mass spectrometer was operated in electron ionization mode (70 eV) with a scan range of ions from 50 to 650 amu. Results of mass spectra were matched with the standard spectrum library of the National Institute of Standards and Technology (NIST).

### Pot Inoculation Experiment of Banana Seedlings

The control efficiency of the strain H3-2 fermentation broth on banana Fusarium wilt disease was evaluated in a greenhouse with an average temperature of 28°C and relative humidity of 70% from March to April in 2021. Foc TR4 overexpressing a green fluorescent protein gene (GFP-Foc TR4) was provided by the Institute of Environment and Plant Protection, China Academy of Tropical Agricultural Sciences, Haikou, China. Banana (“Brazilian,” *Musa acuminata* L. AAA genotype cv. Cavendish) tissue culture seedlings with three to four true leaves were transplanted from seedling bags (one seedling per bag) to a plastic basin (8 cm × 8 cm) containing 1 kg of sterilized soil (Xu et al., 2020). Three treatments were set up as follows: (1) H_2_O (sterile deionized water, negative control); (2) Foc TR4 (positive control, inoculated with GFP-Foc TR4); (3) H3-2 + Foc TR4 (1.0 × 10^7^ CFU ml^–1^ of strain H3-2 fermentation broth and 1.0 × 10^7^ CFU ml^–1^ of GFP-Foc TR4). One hundred milliliters of mixture was added to the roots of banana seedlings as previously described (Wang et al., 2012). Banana seedlings were treated every 7 days. Each treatment contained 30 plants.

### Assessment of Biocontrol Efficiency

After co-inoculation for 35 days, banana roots were collected and sliced using manual operation as thin as possible. The infection and colonization levels of GFP-Foc TR4 were detected immediately using a laser scanning confocal microscope (Olympus FV1000; Olympus, Tokyo, Japan). The excitation spectrum of GFP was set at 488 nm, and autofluorescence of root tissue was set 530 nm.

Plant height, chlorophyll contents of leaves, stem diameter, leaf area, and total dry weight were also measured. A total of 21 plants were selected form each treatment to assess the biocontrol efficiency. Disease symptoms were observed and recorded in each plant. The disease symptoms were classed into five from 0 to 4 as described by Mak et al. (2004). According to the disease class, the disease incidence (DI) was calculated as DI (%) = Σ(Value of class × Number of disease plants in that class)/(4 × Total number of assessed banana seedlings) × 100 according to the previous description (Xu et al., 2020). Biocontrol efficiency (BE) was measured according to the following formula: BE (%) = (DI in Foc TR4 group – DI in H3-2 + Foc TR4 group)/DI in Foc TR4 group × 100.

### Data Analysis

Statistical analysis was performed using the SPSS 22 software (SPSS Inc., Chicago, IL, United States). The difference among treatments was determined using one-way analysis of variance (ANOVA). A Duncan’s multiple range test was applied to determine a significant difference (*p* < 0.05).

## Results

### Isolation of Strain H3-2

**FIGURE 1:**
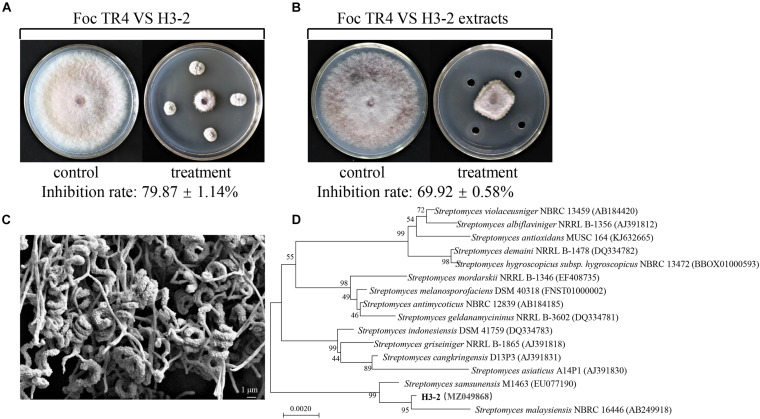
Isolation and identification of strain H3-2 with high antifungal activity against Foc TR4. **(A)** Strain H3-2 inhibiting mycelial growth of Foc TR4. **(B)** Strain H3-2 extracts inhibiting mycelial growth of Foc TR4. **(C)** Morphological characteristics of aerial mycelia and spores of strain H3-2 using SEM. **(D)** Phylogenetic tree of strain H3-2 based on 16S *rRNA* gene sequence analysis. The bootstrap values (%) at the branches were calculated from 1,000 replications.

A total of 44 strains of actinomycetes were isolated from the soil in the banana orchard. Among them, an actinomycete marked with H3-2 was of particular interest because of its potent antagonistic activity against Foc TR4. After dual culture with strain H3-2 for 7 days at 28°C, mycelial diameter of Foc TR4 was 20.0 mm ± 0.08 in comparison to 77.3 mm ± 0.33 in the control (Figure 1A). The inhibition percentage of mycelial growth was 79.9%. Further test on antifungal activity of strain H3-2 extracts showed that mycelial diameter was 29.7 mm ± 0.05 and mycelial inhibition percentage was 69.9% in the treatment group (Figure 1B). Therefore, strain H3-2 was selected for the following study.

### Characteristics and Identification of Strain H3-2

Strain H3-2 grew well on seven different solid media at 28°C for 7 days. The pink soluble pigment was produced on ISP7 medium but was not produced on other media (Supplementary Table 1). The open spirals of spore-bearing mycelia with the revolutions of 3–4 were observed by SEM (Figure 1C). The physiological and biochemical characteristics showed that strain H3-2 exhibited an ability of gelatin liquefaction, can hydrolyze starch and cellulose, but cannot produce H_2_S, urease and xylanase (Supplementary Table 2). It grew normally in the media with pH ranging from 6 to 8 and NaCl content less than 7%. Strain H3-2 could utilize all the tested carbon sources and most of nitrogen sources such as phenylalanine, ammonium sulfate, and potassium nitrate (Supplementary Table 3). These characteristics are typical morphological, physiological, and biochemical features of *Streptomycete*-like organisms. To further confirm phylogenetic relationship between H3-2 and *Streptomyces* spp., 16S *rRNA* sequence of H3-2 was amplified by PCR and sequenced. The sequence exhibited 99.7% similarity with *Streptomyces samsunensis* M1463(T)(EU077190) by alignment against EzBioCloud database and Blast X algorithm in GenBank. A phylogenetic tree constructed by the neighbor-joining method showed that strain H3-2 and *S. samsunensis* M1463(T)(EU077190) clustered into the same group (Figure 1D). Thus, strain H3-2 was identified as *Streptomyces* spp. combining the morphological, physiological, and biochemical characteristics as well as the alignment result of 16S *rRNA* gene sequence.

### Antagonistic Activity of Strain H3-2 Extracts Against Foc TR4

**FIGURE 2:**
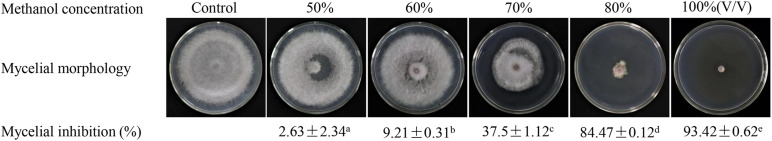
Gradient methanol elution and antifungal activity assay of strain H3-2 extracts. Different lowercase letters represent a significant difference according to the Duncan’s multiple range test (*p* < 0.05).

The crude extracts of strain H3-2 were further extracted with different concentrations of methanol (50–100%, v/v) for measurement of antagonistic activity against Foc TR4. The antagonistic activity of extracts increased significantly along with the increase of methanol concentrations (Figure 2). The inhibition rate of 80% methanol extracts to Foc TR4 was 84.7%. The antifungal activity of 100% methanol extracts was the strongest, and the inhibition rate was 93.4%. Hence, the effect of 100% methanol extracts of strain H3-2 on mycelial growth was selected for further investigations.

### Strain H3-2 Extracts Inhibiting Mycelial Growth of Foc TR4

**FIGURE 3:**
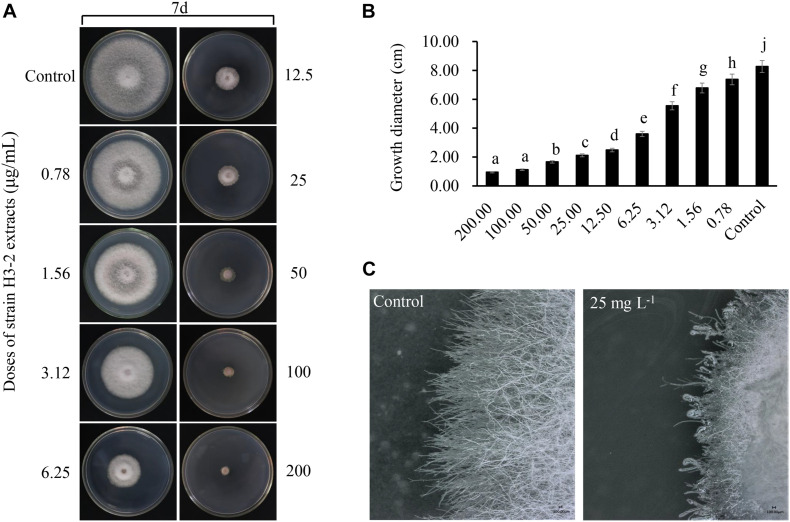
Effects of strain H3-2 extracts on mycelial growth of Foc TR4. **(A)** Mycelial growth of Foc TR4 on PDA medium treated with different concentrations of H3-2 extracts. **(B)** Quantitative analysis of Foc TR4 growth diameters. Different lowercase letters represent a significant difference according to the Duncan’s multiple range test (*p* < 0.05). **(C)** Colonial edge profiles observed using a light microscope. Bar = 100 μm.

Strain H3-2 extracts observably inhibited mycelial growth of Foc TR4 after 7 days. The higher the concentration exhibited the more apparent the inhibition ability (Figure 3A). Compared with the growth diameter (8.27 cm ± 0.06) of Foc TR4 in the control plate, the mycelial growth diameter decreased markedly to 2.48 cm ± 0.12 in the treatment group of 12.5-μg/ml extracts. When the concentration of strain H3-2 extracts reached 200 μg/ml, the colony growth was significantly inhibited and the inhibition rate reached 94.2% ± 0.64 (Figure 3B). In the presence of 25 mg/L extracts, the hyphae at the colony edges were short and thick and did not produce branches (Figure 3C). The toxic regression equation (*y* = 1.1412*x* + 3.9204, *R*^2^ = 0.9922) was further established and the EC_50_ value of strain H3-2 extracts against Foc TR4 was 8.83 μg/ml, which was defined as 1 × EC_50_ in a follow-up study.

#### Effects of Strain H3-2 Extracts on Mycelial Morphological Characteristics of Foc TR4

**FIGURE 4:**
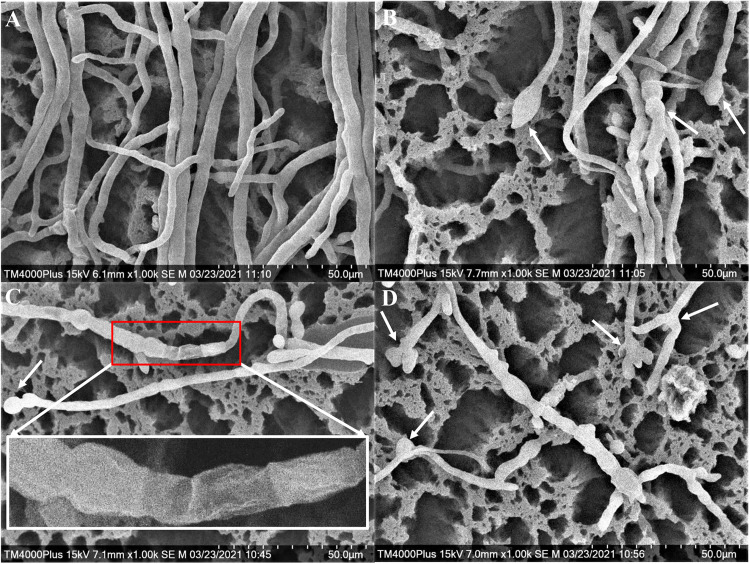
Mycelial morphology of Foc TR4 observed under SEM. **(A)** Mycelial characteristics of Foc TR4 in the absence of stain H3-2 extracts. **(B–D)** Mycelial morphology of Foc TR4 treated with 25 μg/ml of strain H3-2 extracts. The arrows indicate damaged mycelia of Foc TR4 in the presence of strain H3-2 extracts.

The Foc TR4 mycelia collected from the edge of the inhibition zone was different from that of the control by SEM. In the control group, the surface of normal mycelia was smooth and the tops were regular, mellow, and full (Figure 4A). Whereas, the mycelia treated with H3-2 extracts were rough, irregular swelling and cell wall collapsed. The mycelial tops had spherical, fusiform swelling and webbed-foot-shaped branches (Figure 4B–D).

### Effects of H3-2 Extracts on Mycelial Cell Ultrastructure of Foc TR4

**FIGURE 5:**
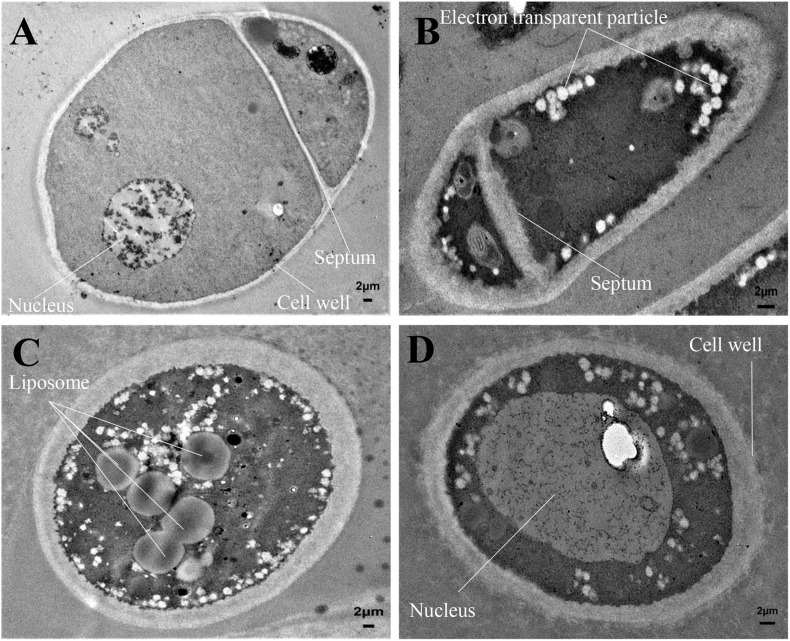
Mycelial cell ultrastructure of Foc TR4 observed under TEM. **(A)** Ultrastructure characteristics of Foc TR4. **(B–D)** Ultrastructure characteristics of Foc TR4 treated with 50 μg/ml of strain H3-2 extracts. The arrows indicate Foc TR4 ultrastructure damaged by strain H3-2 extracts. Bar = 2 μm.

The mycelial cell ultrastructure of Foc TR4 was observed by TEM after treatment with 50 μg/ml of strain H3-2 extracts. In the control group, the cell wall and membrane were intact, and the cytoplasm was regular and uniform (Figure 5A). After treatment with strain H3-2 extracts, the cell wall and septum were thickened and the cell membrane was partly disrupted (Figure 5B). In addition, the increase of liposomes and electron transparent particles was also observed (Figure 5C). The cell nucleus swelled together with chromatin dissolving in the treatment group (Figure 5D).

### Strain H3-2 Extracts Inhibiting Foc TR4 Spore Germination

**FIGURE 6:**
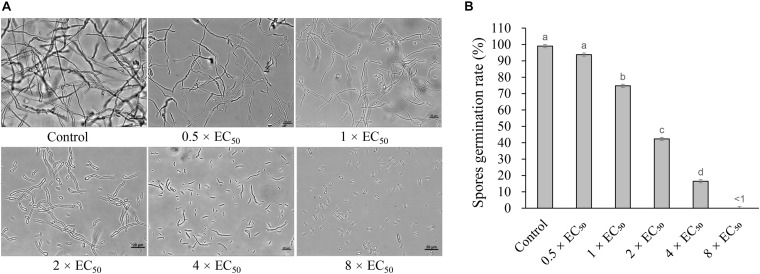
Inhibition of strain H3-2 extracts on spore germination of Foc TR4. **(A)** Spore germination characteristics of Foc TR4 after treatment with 0.5×, 1×, 2×, 4× or 8× EC_50_ extracts. 10% of DMSO was used as a control. Bar = 20 μm. **(B)** The spore germination rate (%) of Foc TR4 after treatment with different dose extracts. Different lowercase letters indicate a significant difference according to the Duncan’s multiple range test (*p* < 0.05).

Strain H3-2 extracts effectively inhibited the spore germination of Foc TR4. The inhibitory efficiency of spore germination was enhanced along with the concentration increase of extracts (Figure 6). Compared with the control group, the spore germination rates of Foc TR4 were 74.8, 42.3, and 16.5% after treatment with 1 × EC_50_, 2 × EC_50_, and 4 × EC_50_ extracts for 12 h, respectively. The germination rate of 0.5 × EC_50_ was not significantly different from that of the control group, but the length of the germ tube was shorter than that of the control group. The spore germination was completely inhibited, and the length of the germ tube significantly failed to develop at the treatment group of 8 × EC_50_ extracts.

### Assay of a Broad-Spectrum Antifungal Activity

**FIGURE 7:**
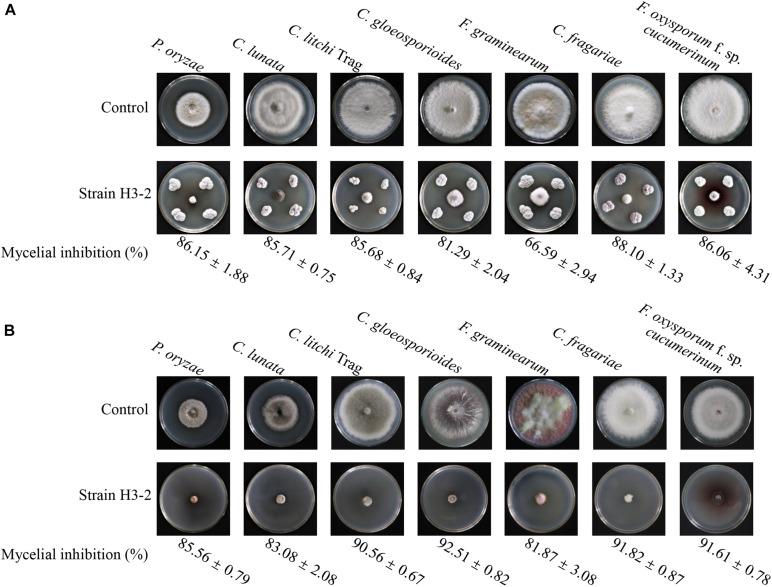
A broad-spectrum antifungal activity of strain H3-2 **(A)** and its extracts **(B)** against the selected seven plant pathogenic fungi.

The strain H3-2 and its extracts showed a broad-spectrum activity against the selected seven phytopathogenic fungi with inhibition rate in the range of 67.0–88.1% and 81.9–92.5%, respectively (Figure 7). The maximal and minimal inhibition activities of stain H3-2 were observed against *C. fragariae* and *F. graminearum*, respectively. For strain H3-2 extracts, the maximum inhibition activity of 92.5% was exhibited against *C*. *gloeosporioides*, and the lowest inhibition activity was 81.9% against *F. graminearum*.

### Chemical Constituent Analysis of Strain H3-2 Extracts by GC-MS

Active chemical constituents of strain H3-2 extracts were analyzed by GC-MS. A total of 14 chemical constituents were identified by alignment of the NIST library based on retention time, molecular mass, match, and the molecular formula (Table 1). The predicted chemical constituents were listed in Supplementary Figure 1, including (1) 4,6-dimethyldodecane, (2) Cyclohexane, octyl-, (3) Undecane, 3,7- dimethyl-, (4) Phenol, 2,4-bis(1,1-dimethylethyl)-, (5) Benzene, 1,2,3-trimethoxy-5-(2-propenyl)-, (6) Hexadecane, (7) Cyclohexane,(4-methylpentyl)-, (8) 9-Heptadecanone, (9) Benzeneprop anoic acid, 3,5-bis(1,1-dimethylethyl)-4- hydroxy-,octadecyl ester, (10) Nonadecane, 2- methyl-, (11) 2-methyloctaco sane, (12) Phenol, 2,2′-methylenebis[6-(1,1-dimethylethyl)-4- methyl-, (13) Hexadecanoic acid, 2-hydroxy-1-(hydroxymethyl) ethyl ester, and (14) Octadecanoi acid, 2-hydroxy-1-(hydroxymethyl)ethyl ester. The peak area represented a quantitative proportion of the predicted compound in the total of extracts.

**TABLE 1:** Chemical constituents in strain H3-2 extracts analyzed by GC-MS.

**No.**	**Predicted compounds**	**Match**	**Probability (%)**	**RT (min)**	**Area (%)**	**Chemical formula**	**Activity**	**References**
1	4,6-dimethyldodecane	774	6.5	16.020	1.93	C_1__4_H_30_	Antimicrobial, antioxidant	Geethalakshmi and Sarada, 2013
2	Cyclohexane, octyl-	879	47.5	19.873	1.63	C_1__4_H_28_	No activity reported	
3	Undecane, 3,7-dimethyl-	770	7.2	20.436	0.7	C_1__3_H_28_	No activity reported	
4	Phenol, 2,4-bis(1,1-dimethylethyl)-	884	45.1	20.769	6.89	C_1__4_H_2__2_O	Inhibitor	Kim et al., 2017
5	Benzene, 1,2,3-trimethoxy-5-(2-propenyl)-	740	37.4	21.332	1.08	C_1__2_H_1__6_O_3_	Antibacterial	Rossi et al., 2007
6	Hexadecane	843	17.7	22.093	0.73	C_1__6_H_34_	No activity reported	
7	Cyclohexane,(4-methylpentyl)-	784	15.5	22.858	1.26	C_1__2_H_24_	No activity reported	
8	9-Heptadecanone	801	79.1	25.213	0.94	C_1__7_H_3__4_O	No activity reported	
9	Benzeneprop anoic acid, 3,5-bis(1,1-dimethylethyl)-4- hydroxy-,octadecyl ester	728	93.2	76.351	12.77	C_3__5_H_6__2_O_3_	Antimicrobial, Anticancer	Elsayed et al., 2020
10	Nonadecane, 2-methyl-	802	13.2	27.635	4.67	C_2__0_H_42_	Antibiofilm	Adnan, 2015
11	2-methyloctaco sane	780	15.4	29.481	2.52	C_2__9_H_60_	No activity reported	
12	Phenol, 2,2′-methylenebis[6-(1,1-dimethylethyl)-4-methyl-	866	95.3	30.268	10.22	C_2__3_H_3__2_O_2_	Insecticidal	Sun et al., 2007
13	Hexadecanoic acid, 2-hydroxy-1-(hydroxymethyl)ethyl ester	786	55.9	31.434	4.79	C_1__9_H_3__8_O_4_	Antioxidant, anti-inflammatory, anthelmintic	Ali et al., 2015
14	Octadecanoi acid, 2-hydroxy-1-(hydroxymethyl)ethyl ester	761	46.1	34.951	4.96	C_2__1_H_4__2_O_4_	No activity reported	

### Strain H3-2 Fermentation Broth on Biocontrol Efficiency of Banana Fusarium Wilt

**FIGURE 8:**
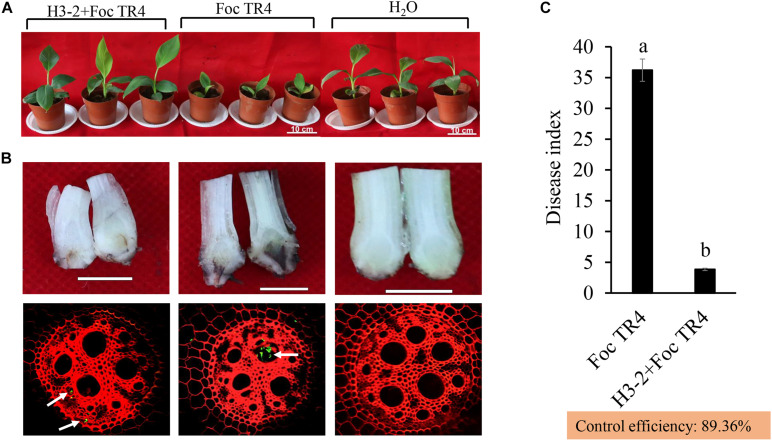
Effects of the strain H3-2 fermentation broth on the growth of banana seedlings and resistance to Foc TR4 in the control (Foc TR4) and the two treatments. **(A)** Chlorotic leaves and growth of banana seedlings were detected 35 days after strain H3-2 treatment. Bars = 10 cm. **(B)** Infection characteristics of Foc TR4 on corms and roots of banana seedlings. The arrows indicate infection sites of Foc TR4. Bars = 1 cm. **(C)** Statistical analysis of disease indexes of banana seedlings 35 days after inoculation with Foc TR4. All experiments were repeated in triplicates. Different lowercase letters indicate a significant difference at the level of *p* < 0.05.

After co-inoculation for 35 days, chlorosis and wilting symptoms appeared in old leaves in the Foc TR4 treatment group, while the banana seedlings had no disease symptoms after treatment with the strain H3-2 fermentation broth (Figure 8A). Compared with the H_2_O and strain H3-2 groups, the growth of banana seedlings was significantly inhibited by Foc TR4. Furthermore, Foc TR4 spores extended from banana root vascular bundles to corms, causing corm rot in the control group. In the strain H3-2 treatment group, no obvious infection occurred in banana corms, and only a few spores of Foc TR4 invaded in the root epidermal cells (Figure 8B). Moreover, the disease index dropped below 1% and the biocontrol efficiency was 89.4% after treatment with H3-2 fermentation broth (Figure 8C).

### Strain H3-2 Fermentation Broth Promoted the Growth of Banana Seedlings

**FIGURE 9:**
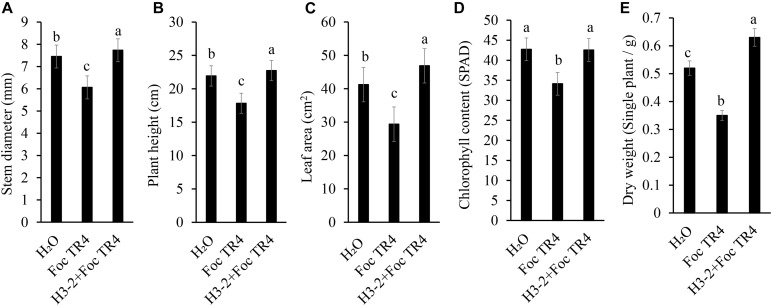
Effect of the strain H3-2 fermentation broth on the growth of banana seedlings. Growth parameters including stem diameter **(A)**, plant height **(B)**, leaf area **(C)**, chlorophyll content **(D)**, and dry weight **(E)** were measured. Different lowercase letters indicate a significant difference at the level of *p* < 0.05.

The growth of banana seedlings was significantly promoted after treatment with the fermentation broth of strain H3-2. Compared with Foc TR4 and H3-2 + Foc TR4 groups, the stem diameter, plant height, leaf area, chlorophyll content and dry weight were increased by 27.6, 27.5, 59.6, 24.7, and 80.0%, respectively (Figures 9A–E). Therefore, the fermentation broth of *Streptomyces* sp. H3-2 showed a growth-promoting role on banana seedlings.

## Discussion

Plant fungal diseases, especially Fusarium wilt, has seriously limited the sustainable development of the banana industry (Li et al., 2021b). Biological control is considered to be high efficiency, broad spectrum, and environment friendly (Chen et al., 2018). Our previous study found that a banana orchard had no disease symptoms of Fusarium wilt for more than 10 years, in which rhizosphere soil was found to contain many functional microorganisms (Zhou et al., 2019). Especially, most of secondary metabolites of *Streptomyces* sp. had a broad-spectrum antifungal activity in controlling plant diseases (Chen et al., 2018; Wei et al., 2020; Li et al., 2021b). In the present study, an actinomycete, named as *Streptomyces* sp. strain H3-2, was isolated from the banana rhizosphere soil. Strain H3-2 and its secondary metabolites effectively inhibit the growth of Foc TR4 and the selected seven phytopathogenic fungi *in vitro*. The EC_50_ value of strain H3-2 extracts against Foc TR4 was 8.83 μg/ml, while EC_50_ values of *Streptomyces* sp. JBS5-6, *Streptomyces* sp. H4, and *Streptomyces* sp. YYS-7 against Foc TR4 were 136.9, 32.6, and 21.1 μg/ml, respectively (Jing et al., 2020; Wei et al., 2020; Li et al., 2021b). Notably, no inhibition activity was observed against endophytic bacteria isolated from banana roots (Supplementary Figure 2), suggesting that *Streptomyces* sp. strain H3-2 has the great potential in controlling banana Fusarium wilt.

Accumulated evidence indicated *Streptomyces* sp. as a biological agent against plant pathogenic fungi mainly because it produced a large number of secondary metabolites with antimicrobial activity (Chen et al., 2018; Yun et al., 2018; Jing et al., 2020; Wei et al., 2020; Li et al., 2021b). *Streptomyces* is also widely used in the medicine industry. More than half of clinical antibiotic drugs and their precursors are produced by *Streptomyces* (Emerson et al., 2012). We found that the inhibition efficiency of extracts isolated using 100% concentration of methanol was the strongest against Foc TR4 (Figure 2). A similar result was demonstrated in our previous studies (Li et al., 2021b). However, extracts of *Streptomyces* sp. JBS5-6 and S*treptomyces* sp. SCA3-4 isolated by ethyl acetate showed more effective antifungal activity (Qi et al., 2019; Jing et al., 2020). It suggested that different kind of compounds were extracted using different solvents, and molecular weights of bioactive metabolites were also distinct in different *Streptomyces* isolates. In this study, inhibitory efficiency was positively correlated with the extract concentration. Mycelial growth was completely inhibited under treatment with more than 200 μg/ml of extracts (Figure 3A). It could be supported that Foc TR4 spores could not germinate after treated with 8 × EC_50_ of extracts. Similar results showed that spore germination of *F. oxysporum* was inhibited by extracts of *Streptomyces* sp. WP-1 (Peng et al., 2020).

In addition, the colonial tops of Foc TR4 treated with strain H3-2 extracts became swelling and the mycelia abnormally branched. Similarly, abnormal morphology of *F. oxysporum* fungal mycelia were observed after treatment with extracts of *Streptomyces* sp. JBS5-6 (Jing et al., 2020). Extensive degradation of the cell wall and membrane was also observed, and some cellular contents gradually disappeared. This finding supported the phenomenon of the increase of liposomes and formation of transparent electronic density area. Antifungal ability of *Streptomyces* may be involved in nutrient competition, degradative enzyme biosynthesis, response of fungal defenses, antibiosis, nitrous oxide production, and quorum quenching (Cohen and Mazzola, 2006; Dan et al., 2010). However, the underlying mechanisms of strain H3-2 against Foc TR4 require further studies.

Using GC-MS, some important secondary metabolites of antifungal activity of strain H3-2 were identified. Two phenolic compounds, phenol,2,4-bis(1,1-dimethylethyl)- and phenol,2,2′-methylenebis[6-(1,1-dimethylethyl)-4-methyl- were detected in strain H3-2 extracts, which were reported as antimicrobial agents due to its ability to remove free radicals (Pintać et al., 2019). Recently, phenolic compounds extracted from grape leaves had an obvious inhibitory effect on oral microorganisms, such as *S. aureus*, *E. coli*, and *S. mutans* (Moldovan et al., 2020). In addition, the ester compounds were also detected in our study. The highest level of benzeneprop anoic acid,3,5-bis(1,1-dimethylethyl)-4- hydroxy-,octadecyl ester in the GC-MS fractions showed antimicrobial and anticancer activity (Elsayed et al., 2020). Hydrocarbon exhibited antagonistic ability on various pathogens (Mothana et al., 2011). The alkane (4,6-dimethyldodecane) and olefin (benzene,1,2,3- trimethoxy-5-(2-propenyl)-) detected in the present study have been reported to have antimicrobial activity (Rossi et al., 2007; Geethalakshmi and Sarada, 2013). Some acid compounds also showed antimicrobial activity (Himaman et al., 2016). Hexadecanoic acid,2- hydroxy-1-(hydroxymethyl) ethyl ester extracted from medicinal plants (*Melia azedarach* leaves) had antioxidant, anti-inflammatory, and anthelmintic activities (Ali et al., 2015). Thus, we speculated that these compounds play a key role in the broad-spectrum antifungal activity of *Streptomyces* sp. H3-2 (Figure 7).

The pot inoculation experiment demonstrated strong biocontrol efficiency of *Streptomyces* sp. H3-2 on banana Fusarium wilt disease. Notably, the fermentation broth of *Streptomyces* sp. H3-2 also significantly promoted the growth of banana seedlings including stem diameter, plant height, leaf area, chlorophyll content, and dry weight in comparison to the Foc TR4 treatment group. Similarly, *Streptomyces* sp. FS-4 not only significantly reduced banana Fusarium wilt, but also promoted the growth of banana seedlings (Duan et al., 2020). Rungin et al. (2012) reported that *Streptomyces* sp. GMKU 3100 stimulated the growth of rice and mungbean plants by producing siderophores. Verma et al. (2011) screened three *Streptomyces* sp. strains promoting plant growth and inhibiting the growth of *Alternaria alternata*, a causal agent of early blight disease in tomato plants. Although the application of *Streptomyces* in field trials was limited due to the complex external environments, antifungal activity of *Streptomyces* sp. H3-2 was confirmed in both pot inoculation experiment and plate culture experiment. Our previous study found that the inhibition activity stability of *Streptomyces* extracts can be kept to some extent under different treatments with temperature, pH, and ultraviolet (UV) (Li et al., 2021a). In addition, *Streptomyces* sp. H3-2 isolated from tropical and subtropical regions with high temperature and strong UV has proven its ability to overcome the complex growth environments, like other species and of *Streptomyces*. Hence, antifungal activity of *Streptomyces* sp. H3-2 may be more stable in agriculture application.

## Conclusion

*Streptomyces* sp. H3-2 exhibits a strong antifungal activity against Foc TR4 and its extracts and effectively inhibited the mycelial growth and spore germination of Foc TR4. Mycelial cells of Foc TR4 become deformed and ultrastructure disappeared after treatment with strain H3-2 extracts. Strain H3-2 and its crude extracts exhibited broad-spectrum antifungal activity against the tested seven phytopathogenic fungi. Moreover, the fermentation broth of strain H3-2 significantly improved plant resistance to Foc TR4 infection and promoted the growth of banana seedlings. Fourteen chemical constituents in strain H3-2 extracts were identified with most of them having antimicrobial activity. Our study demonstrated that *Streptomyces* sp. strain H3-2 has the potential as a biocontrol agent for preventing banana plants from the infection of Foc TR4.

## Data Availability Statement

The raw data supporting the conclusions of this article will be made available by the authors, without undue reservation.

## Author Contributions

NZ, MW, DZ, WW, and JX developed the ideas and designed the research plans. MW, WW, and JX supervised the research work and provided funding support. NZ, DZ, PL, and YuC were involved in soil sampling, observation of SEM, and pot experiment of banana seedlings. NZ, DZ, MW, WW, and JX analyzed the data. NZ, MW, and WW prepared the manuscript. YiC helped in revision. All authors contributed to the article and approved the submitted version.

## Conflict of Interest

The authors declare that the research was conducted in the absence of any commercial or financial relationships that could be construed as a potential conflict of interest.

## Publisher’s Note

All claims expressed in this article are solely those of the authors and do not necessarily represent those of their affiliated organizations, or those of the publisher, the editors and the reviewers. Any product that may be evaluated in this article, or claim that may be made by its manufacturer, is not guaranteed or endorsed by the publisher.

## Footnotes

1
http://www.ncbi.nlm.nih.gov/BLAST

2
https://www.ezbiocloud.net/identify

## Supplementary Material

Supplementary Figure 1 Total ion current chromatograms of strain H3-2 extracts.

Supplementary Figure 2 Symbiotic experiment of strain H3-2 with endophytic bacteria isolated from banana roots. Different numbers below the pictures represent the diverse endophytic bacteria. Strain H3-2 is first cultured on the YE medium for 2 days, and then each endophytic bacterium is inoculated using a cross method. After co-culture for 24 h, the symbiotic result is observed. The experiment is performed in triplicates.

Supplementary Table 1 Growth characteristics of strain H3-2 on different solid culture media.

Supplementary Table 2 Physiological and biochemical characteristics of strain H3-2.

Supplementary Table 3 Carbon and nitrogen source utilization of strain H3-2.
