# Supplementary material for: A Novel Antifungal Actinomycete Streptomyces sp. Strain H3-2 Effectively Controls Banana Fusarium Wilt
Source: Front Microbiol. 2021 Aug 23;12:706647. doi: 10.3389/fmicb.2021.706647 (PMC8419470; doi:10.3389/fmicb.2021.706647)
Supplement: Supplementary Figure 1 — Total ion current chromatograms of strain H3-2 extracts. [file Data_Sheet_1.docx]

Supplementary Material

# Supplementary Tables

**TABLE S1 | Characteristics of strain H3-2 on different solid culture media**

| **Culture medium** | **Aerial**  **Hyphae** | **Vegetative Mycelium** | **Soluble Pigment** | **Colony Characteristics** | **Growth Conditions** | **Single Colony** | **Mycelium** |
| --- | --- | --- | --- | --- | --- | --- | --- |
| ISP2 | White | Yellow | None | Dry and wrinkled | +++ | 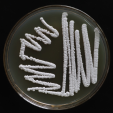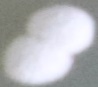 | |
| ISP3 | Grey | Dark-Grey | None | Dry, micropore and wrinkled | +++ | 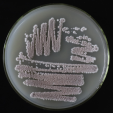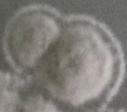 | |
| ISP4 | Grey-White | Light-Gray | None | Moist, micropore and wrinkled | ++ | 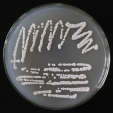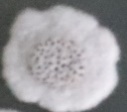 | |
| ISP5 | Grey-White | Creamy White | None | Dry, hard and wrinkled | + | 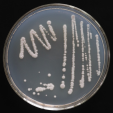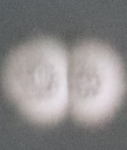 | |
| ISP6 | Yellow-Grey | Off-White | None | Dry, hard and granular sensation | ++ | 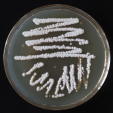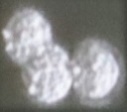 | |
| ISP7 | Steel-Gray | Light-Brown | Pink | Dry, hard and granular sensation | + | 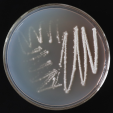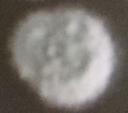 | |
| Gause’s no.1 | Light Steel-Gray | Grey | None | Dry, wrinkled and micropore | ++ | 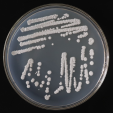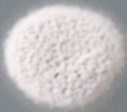 | |


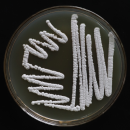

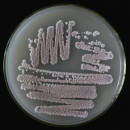

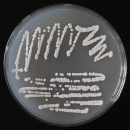

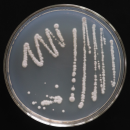

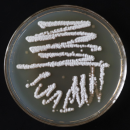

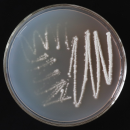

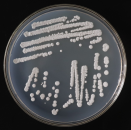

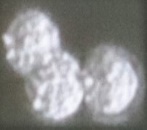

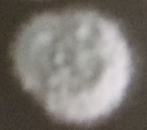

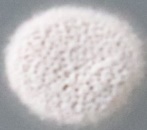

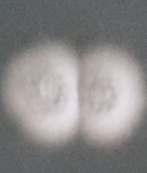

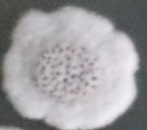

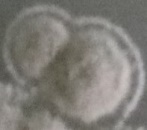

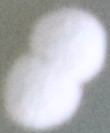


**TABLE S2 | Physiological and biochemical characteristics of strain H3-2**

| Physiological and biochemical experiment | Result |
| --- | --- |
| **Physiological test** |  |
| pH tolerance | 6-8 |
| Optimum growth pH | 6 |
| NaCl tolerance (%) | Not more than 7% |
| **Biochemical test** |  |
| Twain -20 | + |
| Twain -40 | + |
| Twain -80 | + |
| Methyl red test | - |
| Voges-Proskauer test | - |
| Gelatin liquefaction | + |
| H_2_S production | - |
| Starch hydrolysis | + |
| Cellulose hydrolysis | + |
| Urease production | - |
| Xylanase hydrolysis | - |

Note: “+” is indicates positive; “-” is indicates negative.

**TABLE S3 | Carbon and nitrogen source utilization of strain H3-2**

| **Carbon test items** | **Result** | **Nitrogen test items** | **Result** |
| --- | --- | --- | --- |
| D-Melezitose | + | L-glutamic acid | - |
| L-Rhamnose | ++ | Phenylalanine | + |
| Tryptone | +++ | L-tyrosine | + |
| Soluble starch | ++ | Anhydrous amino acid | ++ |
| Glucose | + | α- naphthenic acid | - |
| Raffinose | + | Cysteine | ++ |
| D-Fructose | + | L-asparagine | ++ |
| D-Ribose | + | Arginine | ++ |
| D-Cellobiose | + | Histidine | ++ |
| D-Galactose | + | Diammonium hydrogen Citrate | - |
| Sucrose | + | Potassium nitrate | ++ |
| Sorbose | ++ | Salicin | + |
| Inositol | ++ | Ammonium sulphate | + |
| Trehalose | ++ | Ammonium ferric citrate | ++ |

Note: “+++”is indicates growth in medium in good; “++”is indicates growth in medium in general; “+” is indicates growth in medium in weak; “-” is indicates no growth in medium

# Supplementary Figures


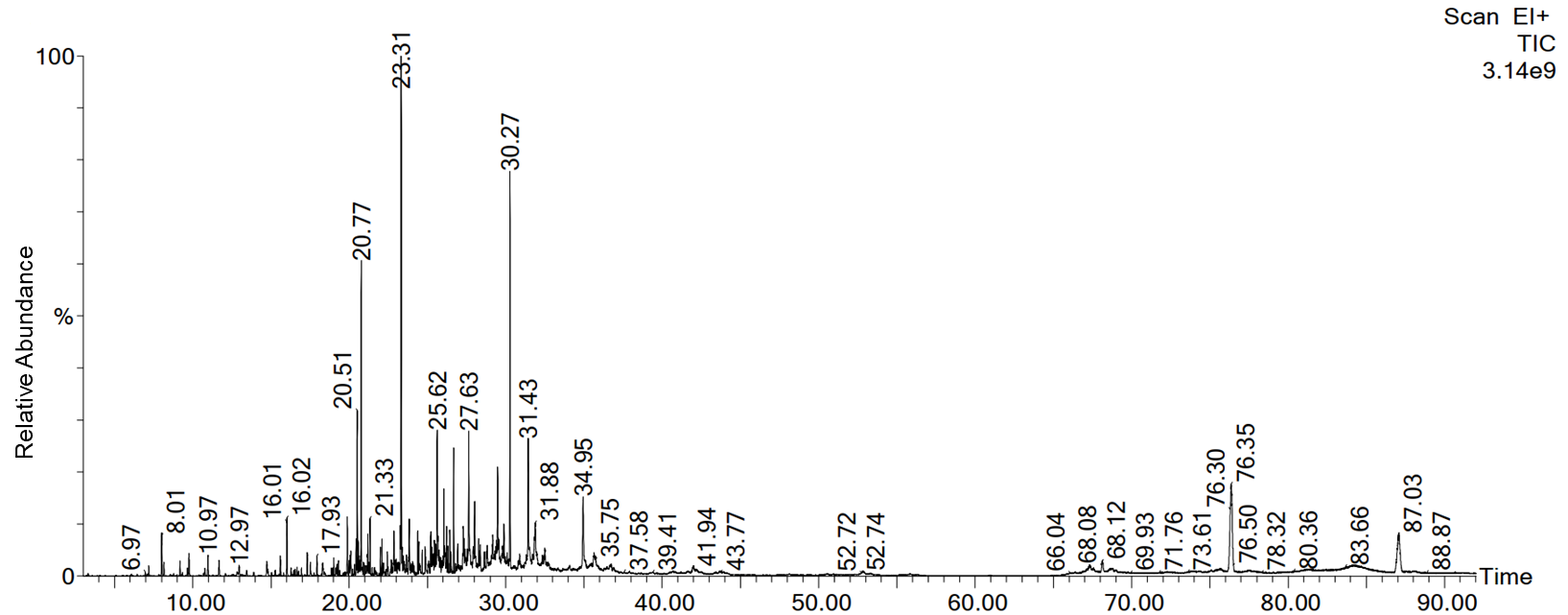


**FIGURE S1 |** Total ion current chromatograms of strain H3-2 extracts


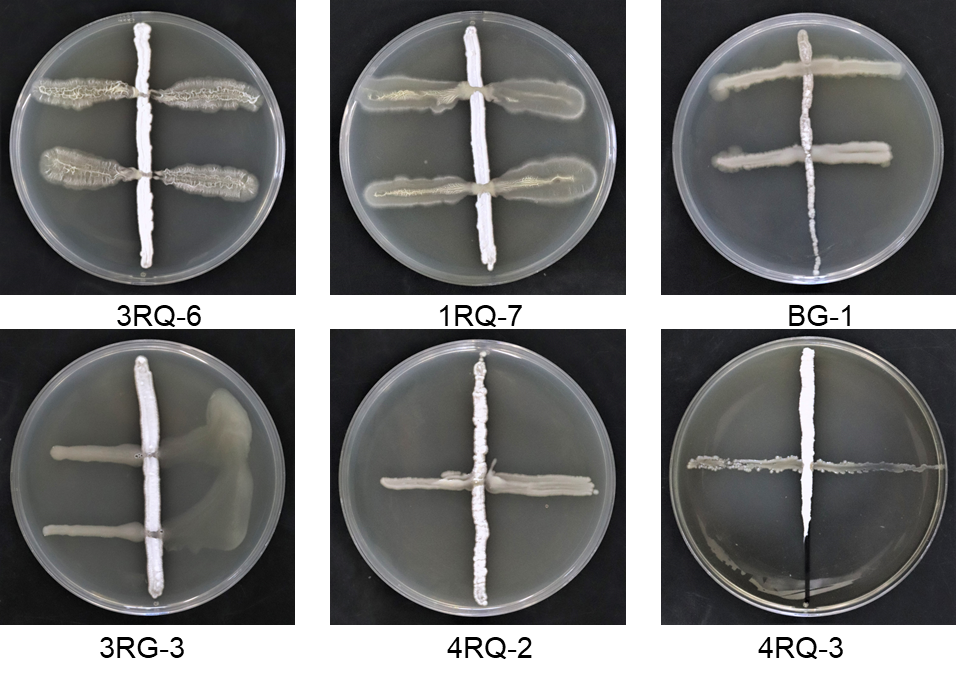


**FIGURE S2 |** Symbiotic experiment of strain H3-2 with endophytic bacteria isolated from banana roots. Different numbers below the pictures represent the diverse endophytic bacteria. Strain H3-2 is first cultured on the YE medium for 2 days, and then each endophytic bacterium is inoculated using a cross method. After co-culture for 24 hours, the symbiotic result is observed. The experiment is performed in triplicates.
